# Benthic N_2_ fixation in coral reefs and the potential effects of human-induced environmental change

**DOI:** 10.1002/ece3.1050

**Published:** 2014-03-31

**Authors:** Ulisse Cardini, Vanessa N Bednarz, Rachel A Foster, Christian Wild

**Affiliations:** 1 Coral Reef Ecology Group (CORE), Leibniz Center for Tropical Marine Ecology (ZMT)Fahrenheitstr. 6, Bremen, 28359, Germany; 2 Max Planck Institute for Marine MicrobiologyCelsiusstr. 1, Bremen, 28359, Germany; 3 Faculty of Biology and Chemistry (FB 2), University of BremenBremen, Germany

**Keywords:** Coral reefs, cyanobacteria, deoxygenation, diazotrophs, eutrophication, global warming, dinitrogen fixation, ocean acidification, symbiosis, ultraviolet radiation stress

## Abstract

Tropical coral reefs are among the most productive and diverse ecosystems, despite being surrounded by ocean waters where nutrients are in short supply. Benthic dinitrogen (N_2_) fixation is a significant internal source of “new” nitrogen (N) in reef ecosystems, but related information appears to be sparse. Here, we review the current state (and gaps) of knowledge on N_2_ fixation associated with coral reef organisms and their ecosystems. By summarizing the existing literature, we show that benthic N_2_ fixation is an omnipresent process in tropical reef environments. Highest N_2_ fixation rates are detected in reef-associated cyanobacterial mats and sea grass meadows, clearly showing the significance of these functional groups, if present, to the input of new N in reef ecosystems. Nonetheless, key benthic organisms such as hard corals also importantly contribute to benthic N_2_ fixation in the reef. Given the usually high coral coverage of healthy reef systems, these results indicate that benthic symbiotic associations may be more important than previously thought. In fact, mutualisms between carbon (C) and N_2_ fixers have likely evolved that may enable reef communities to mitigate N limitation. We then explore the potential effects of the increasing human interferences on the process of benthic reef N_2_ fixation via changes in diazotrophic populations, enzymatic activities, or availability of benthic substrates favorable to these microorganisms. Current knowledge indicates positive effects of ocean acidification, warming, and deoxygenation and negative effects of increased ultraviolet radiation on the amount of N fixed in coral reefs. Eutrophication may either boost or suppress N_2_ fixation, depending on the nutrient becoming limiting. As N_2_ fixation appears to play a fundamental role in nutrient-limited reef ecosystems, these assumptions need to be expanded and confirmed by future research efforts addressing the knowledge gaps identified in this review.

## Introduction

In the decades to come, life in the ocean will be confronted with a series of environmental conditions that have no parallel in human history (Harnik et al. 2012). Understanding and predicting the effects of human-induced climate change on marine ecosystems and the organisms within is therefore a current research priority (Garrard et al. 2012; Rees 2012; Salihoglu et al. 2012). Of particular concern are the effects of environmental change on marine microbes as microorganisms drive the elemental transformations of the biogeochemical cycles in the oceans and on land (Gruber 2011).

**Figure 1:**
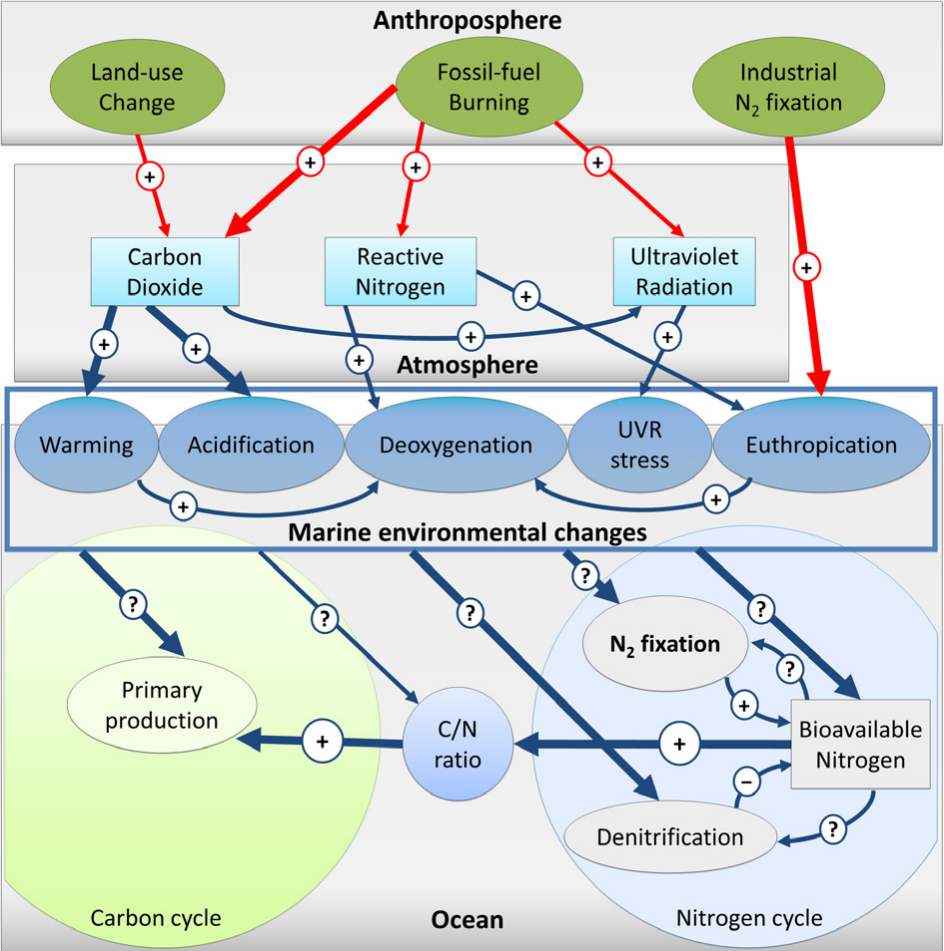
Nitrogen–carbon–climate interactions. Shown are the main interacting drivers during the Anthropocene. Signs indicate an increase (+) or a decrease (−) in the factor shown; (?) indicate an unknown impact. Colors of the arrow indicate direct anthropogenic impacts (red) or natural interactions (blue, many of which also modified by human influence). Strength of the interaction is expressed by the arrow thickness. Only selected interactions are represented. Adapted from Gruber and Galloway (2008).

The marine nitrogen (N) cycle is one of the most important of all biogeochemical cycles, as N is an essential building block in all life forms. The N cycle significantly influences the cycles of other elements and particularly the carbon (C) cycle (Fig. 1), because N is considered the most limiting element for biological productivity in the open sea (Gruber 2008; Canfield et al. 2010). Research projects worldwide have focused on the N cycle and investigated the main consequences of human alteration as a result of the production and industrial use of synthetic nitrogen fertilizers (e.g., (Galloway et al. 2003, 2004, 1995). This resulted in an improved understanding of the consequences of the anthropogenic N problem (Galloway et al. 2004). On the contrary, less studied are the interactions of N with the C cycle and their consequences for the climate, particularly in the context of the increasing human interferences in the Earth system (Falkowski et al. 2000; Gruber and Galloway 2008). Indeed, understanding the N–C–climate interactions (Fig. 1) is becoming increasingly pressing as the release of carbon dioxide (CO_2_) from the burning of fossil fuels is dramatically changing the world's climate (IPCC 2007).

The two main biological processes of the marine N cycle are N gain (i.e., dinitrogen (N_2_) fixation, the conversion of N_2_ to organic N) and N loss (i.e., denitrification, the conversion of nitrate to N_2_, and anammox, anerobic ammonium oxidation). These are particularly important because of the inability of most marine organisms to use elemental N_2_ (i.e., dissolved N gas, the most abundant chemical form). As a consequence, their balance determines the net biologically available N for the biosphere and therefore marine productivity (Arp 2000; Gruber 2005, 2008). Nevertheless, producing a balanced marine N budget has been difficult, with a large apparent deficit (∼200 Tg·N·year^−1^) in the oceanic N_2_ fixation rate compared to N loss (Mahaffey et al. 2005; Codispoti 2007; Deutsch et al. 2007), and it is still a matter of great debate (Großkopf et al. 2012).

Biological N_2_ fixation can only be carried out by some prokaryotes, including a small but diverse group of bacteria and archaea commonly referred to as diazotrophs (Zehr et al. 2003; Kneip et al. 2007). The preferred ecological niche of diazotrophs was assumed to be largely limited to the open ocean oligotrophic gyres that are typically characterized by high light intensities, high O_2_ concentrations, and low N availabilities (Karl et al. 2002). However, recent research on the phylogenetic diversity and distributions of *nifH* (the functional gene which encodes for nitrogenase, the enzyme responsible for N_2_ fixation) found N_2_-fixing microorganisms throughout all marine environments, ranging from deep-sea vents to highly productive shelf areas (Mehta et al. 2003; Zehr et al. 2003; Dekas et al. 2009; Farnelid et al. 2011; Fernandez et al. 2011; Hamersley et al. 2011).

Shallow coral reef environments are also recognized as major contributors of new N into the oceans, likely supporting a major fraction of total benthic N_2_ fixation on a global scale (O'Neil and Capone 2008). However, these ecosystems are highly vulnerable and face an uncertain future as they are exposed to multiple increasing anthropogenic disturbances such as global warming, ocean acidification, increasing ultraviolet (UV) exposure, sea level rise, eutrophication, pollution, overfishing, and shoreline development (Hughes et al. 2010; Pandolfi et al. 2011; Frieler et al. 2013).

Global climate change and other anthropogenic pressures have the potential to not only alter the physiology of reef organisms directly (Hoegh-Guldberg et al. 2007; De'ath et al. 2009; Kleypas and Yates 2009), but also indirectly through impacts on reef-associated microorganisms (Vega Thurber et al. 2009; Meron et al. 2011; Witt et al. 2011). These invisible players are important drivers of coral reefs (Barott and Rohwer 2012). In fact, as microorganisms are the fastest in reacting to disturbances and their responses are often nonlinear, they are able to provide near real-time trajectories for a coral reef (Barott and Rohwer 2012). Therefore, their study is fundamental for understanding the functioning of the entire ecosystem in the face of climate change. Finally, as we come to understand the implications of the increasing anthropogenic pressure on coral reef environments, it is important that we evaluate impacts on major biogeochemical processes, and N_2_ fixation is an obvious key ecological process that requires such evaluation.

Several publications recently attempted to review the current literature on N_2_ fixation. However, these have been focused primarily on the open ocean (Karl et al. 2002; Mahaffey et al. 2005; Sohm et al. 2011b; Zehr 2011), on recent discoveries of previously unknown or less studied symbiotic associations with diazotrophs (Foster and O'Mullan 2008; Fiore et al. 2010), or on the *nifH* gene diversity and distribution (Zehr et al. 2003; Riemann et al. 2010), while only a few focused on coral reefs (Capone 1996; Carpenter and Capone 2008; O'Neil and Capone 2008). Moreover, these latter reports have mainly concentrated on the N cycle as a whole and particularly on the contribution of fixed N to the global ocean by coral reef habitats. However, recent findings stress the importance of specific associations between benthic reef organisms and N_2_ fixers and furthermore identify the potential effects of climate change. Therefore, our primary foci are to review the present state of knowledge on benthic N_2_ fixation in coral reefs and to provide a descriptive overview of the symbioses between benthic reef organisms and diazotrophs. Additionally, we will review the current knowledge on the effects of several anthropogenic impacts on the biological process of N_2_ fixation. However, due to the lack of information available with regard to the effects of environmental conditions on benthic reef N_2_ fixation, reference to the more studied pelagic realm will be given in the text when appropriate for comparison. Finally, we will provide a baseline upon which future coral reef research can build, suggesting some key research questions to be addressed and promising methodologies to be applied which may help to shed light on this crucial reef biogeochemical process.

## N_2_ Fixation in Coral Reef Ecosystems

### Reef productivity

The study of relationships between C and nutrient fluxes is central to understanding material and energy fluxes in coral reefs, which ultimately set limits to metabolic performance of the ecosystem (Atkinson 2011). Although coral reefs show the highest rates of gross primary productivity worldwide, their existence is generally associated with waters that are very low in the nutrients necessary for primary production, akin to “oases in a marine desert” (Hoegh-Guldberg 1999). This conundrum is generally known as the reef paradox, sometimes called the Darwin's paradox because he was the first to notice it. Remarkably different is their estimated net community production (i.e., the difference between gross primary production and respiration), with a net gain (or loss) of matter within the system which is approximately zero (Gattuso et al. 1998).

The high biomass and gross productivity of these ecosystems is therefore explained by a tight internal recycling of matter that primarily occurs in the benthos (O'Neil and Capone 2008). Indeed, most of the organic matter produced on the reef is recycled and retained in living organisms or sediments within the reef system (Suzuki et al. 1995; Wild et al. 2004). Reef productivity cannot be sustained through the limited input of nutrients from the surrounding oceans, although sometimes these can be supplied by upwelling or internal tidal bores (Gattuso et al. 1998) as well as by nutrient advection or loading from land (Lapointe et al. 2004; Alongi and McKinnon 2005). Therefore, a thriving coral reef needs a finely tuned microbial-driven system to capture and recycle the nutrients necessary to support primary production (Garren and Azam 2012). On the other hand, if only regenerated nutrients were available, gross photosynthesis could not exceed respiration, unless a change in elemental ratios occurred: net growth and net export require the input of new N into the system (Szmant-Froelich 1983).

### Benthic N_2_ fixation as a primary N source

In the past, benthic N_2_ fixation was considered the main source of “new” N in the World's oceans (Capone and Carpenter 1982). However, actual estimates suggest a much greater contribution of pelagic N_2_ fixation (a substantial fraction can be attributed to the colonial filamentous free-living cyanobacterium *Trichodesmium* spp.) compared to the amount of N that is fixed annually by benthic N_2_ fixers (Gruber 2004). Recent research showed that diazotrophs in the smaller size fraction (<10 *μ*m cell diameter) are likely an equally important source of new N in the open ocean (Zehr et al. 2001; Mazard et al. 2004; Montoya et al. 2004; Needoba et al. 2007; Moisander et al. 2010). Moreover, several have identified that rates of N_2_ fixation are often underestimated because of current methodological approaches (Mohr et al. 2010; Wilson et al. 2012). However, benthic N_2_ fixation estimates are based on old studies, mostly snapshots of particular benthic environments extrapolated to much larger areas. Therefore, a re-evaluation is necessary, because we are only beginning to understand the extent and importance of benthic marine N_2_ fixation. In general, the seafloor hosts a wide diversity of geological and ecological settings supporting unique microbiological and faunal communities that might greatly contribute to the global input of fixed N (e.g., Dekas and Orphan (2011). In particular, benthic N_2_ fixation assumes an overwhelming role in those ecosystems whose primary production is strongly N-limited and which are surrounded by highly N-depleted oceanic waters, such as coral reefs. Indeed, several coral reef studies observed export of N in the form of nitrate (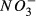), dissolved organic (DON), and particulate organic nitrogen (PON) in excess of inputs (Webb et al. 1975; Smith 1984; Suzuki and Casareto 2011), implying a source of fixed N from within the reef community, which can be attributed to N_2_ fixation.

Introductions of bioavailable N through N_2_ fixation can increase rates of primary production (Dugdale and Goering 1967), and low *δ*^15^N signatures noted in several reef primary producers is consistent with the hypothesis that much of the N in reef systems is derived from N_2_ fixation (Yamamuro et al. 1995; Hilting et al. 2013). However, N_2_ fixation in coral reef environments remains underinvestigated and likely underestimated (O'Neil and Capone 2008).

## Distribution and Abundance of Diazotrophs on Coral Reefs

### Epibenthic diazotrophs

Epibenthic biofilms on solid surfaces are present everywhere in the aquatic environment. In particular, biofilms growing on living organisms may affect the fluxes of information, chemical signals, energy, nutrients, and matter across the host's body surface. Therefore, biofilms have an important ecological role in controlling the abiotic and biotic interactions of the host (Wahl et al. 2012).

**Figure 2:**
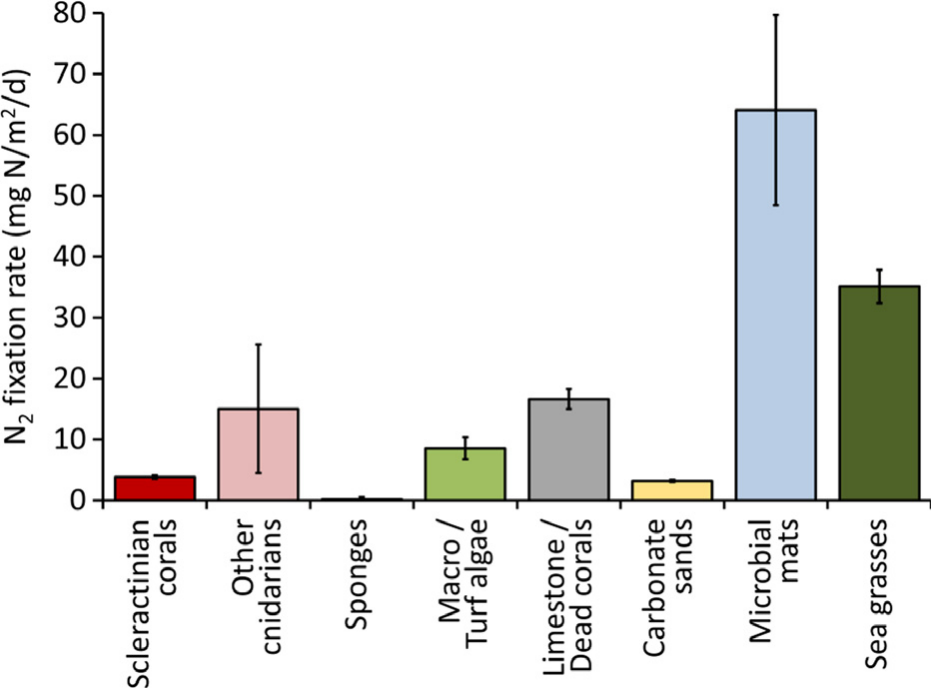
Contribution of the main benthic coral reef components to the input of new N in the reef via N_2_ fixation. Rates (average ± SE) were obtained from the available studies which reported nitrogenase activity associated with benthic reef organisms and substrates normalized to surface area. A list of the literature used is available in Table S1.

In coral reef ecosystems, studies on benthic N_2_ fixation studies have largely focused on microbial mats (Charpy-Roubaud et al. 2001; Steppe et al. 2001; Charpy-Roubaud and Larkum 2005; Charpy et al. 2007, 2012a,b). These are dominated by cyanobacteria, which are found associated with sulfur bacteria and other microorganisms (Charpy et al. 2012a). They form flat, extensive mats, several millimeters thick on sand and limestone. These bacterial mats show the highest rates of N_2_ fixation when compared to all the other main reef benthic components (Fig. 2). Charpy-Roubaud et al. (2001) found that N_2_ fixation associated with reef lagoon sediments, limestone surfaces, and particularly cyanobacterial mats could account for about 25% of the N demand of benthic primary production in a coral atoll in French Polynesia. A follow-up study investigated the reef rim at the same location and found similarly high areal rates largely associated with cyanobacterial mat communities accounting for about 28% of N_2_ fixation of the entire lagoon (Charpy-Roubaud and Larkum 2005).

N_2_ fixation in sea grass meadows have also been extensively studied in tropical coral reef areas (Patriquin and Knowles 1972; McRoy et al. 1973; Capone et al. 1979; Capone and Taylor 1980; O'Donohue et al. 1991; Moriarty and O'Donohue 1993; Blackburn et al. 1994; Welsh 2000; Hamisi et al. 2009), showing rates comparable to those observed in cyanobacterial mats (Fig. 2). Although some studies mainly attributed the measured nitrogenase activity to the epiphytic cyanobacteria on the leaves, often high activities were found associated with the root systems and the rhizosphere sediments, where phototrophic organisms could be outcompeted (Welsh 2000). Patriquin and Knowles (1972) argued that heterotrophic bacteria within rhizosphere sediments were providing most of the fixed N requirements in three different sea grass meadows from Barbados. Later observations demonstrated the importance of sulfate-reducing bacteria (SRBs) in rhizosphere N_2_ fixation (Capone 1982; McGlathery et al. 1998) and that an appreciable fraction of the energy derived from sulfate reduction supported N_2_ fixation (Welsh et al. 1996a,b,c; Nielsen et al. 2001).

N_2_ fixation activity also occurs on limestone surfaces, coral rubble, and coral skeletons (Fig. 2)(Crossland and Barnes 1976; Larkum 1988; Shashar et al. 1994a,b; Davey et al. 2008). These “bare” substrates are typically omnipresent on coral reefs, but their contribution to the total benthic cover is strongly dependent on the hydrodynamics and sedimentation rate. High rates of nitrogenase activity have been found in coral rubble from the Red Sea (Shashar et al. 1994b) and limestone substrates from the Great Barrier Reef (Larkum et al. 1988). Moreover, skeletons of coral which had undergone thermal bleaching showed high associated nitrogenase activity, with rates up to 30 times greater than those measured on live corals (Davey et al. 2008).

N_2_ fixation has been further identified in bacterial epiphytes on benthic reef macroalgae (Capone et al. 1977; France et al. 1998; Koop et al. 2001) as well as associated with algal turfs (Fig. 2) (Williams and Carpenter 1997, 1998). N_2_-fixing cyanobacteria were among the dominant active members of the microbial community associated with a red alga of the broadly distributed genus *Laurencia* (de Oliveira et al. 2012). Active N_2_ fixers are associated with members of the green algal genera *Caulerpa* (Williams et al. 1985; Chisholm and Moulin 2003) and *Codium* (Rosenberg and Paerl 1981). Both these algae are common on sandy and rocky reef substrates in intertidal and subtidal zones of tropical and subtropical coastal waters throughout the world. *Caulerpa taxifolia* appears to enhance N_2_ fixation by releasing photosynthetic products into the rhizosphere (Chisholm and Moulin 2003). The excreted organic C, consumed by fermenting bacteria, creates substrate and strong reducing conditions that are favorable to N_2_ fixation by SRBs. This process enhances organic matter turnover and nutrient supply to the alga's rhizoids, assisting this species to proliferate upon refractory organic sediments in low-nutrient seawater (Chisholm and Moulin 2003).

Active diazotrophs are also found associated with the ubiquitous reef carbonate sediments (Wilkinson et al. 1984; Corredor and Morell 1985; O'Neil and Capone 1989; Capone et al. 1992; Miyajima et al. 2001; Hewson and Fuhrman 2006; Werner et al. 2008), where N_2_ fixation can account for substantial N flow into the system (Capone et al. 1992). In fact, rates of N_2_ fixation in nonvegetated reef sediments are one order of magnitude lower than in cyanobacterial mats (Fig. 2) (Burris 1976; Iizumi and Yamamuro 2000; Bauer et al. 2008), but, when extrapolated over the entire reef area covered by mobile substrates, they can make a significant contribution to the overall coral reef N budget (Capone 1996; O'Neil and Capone 2008).

### Symbiotic associations

Interest in marine microbial symbioses is growing rapidly because of the increasing awareness of the vast range of animal–bacterial interactions that is fundamentally altering our understanding of animal biology (McFall-Ngai et al. 2013). Symbioses have the potential to increase the fitness of the host and are implicated in its metabolism and growth, chemical defense production, as well as its susceptibility to biotic and abiotic stressors (Erwin et al. 2012). Specifically, several benthic organisms have coevolved nutritional mutualisms with diazotrophic bacteria in N-limited environments such as coral reefs (Fiore et al. 2010).

Symbiotic cyanobacteria and bacteria are found in almost all marine sponges (Carpenter and Foster 2002; Thacker 2005; Webster and Taylor 2012) where the processes of N_2_ fixation, nitrification, denitrification, and anammox were all reported to occur (Wilkinson and Fay 1979; Diaz and Ward 1997; Wilkinson 1999; Mohamed et al. 2008, 2009; Hoffmann et al. 2009; Schläppy et al. 2010). However, the study of the ecological significance of diazotrophic symbionts in sponges and their contribution to the reef N budget has proven difficult (Wilkinson 1999). Rates shown in Fig. 2 are taken from the only study, to our knowledge, which has reported values normalized to surface area (Shashar et al. 1994b). Moreover, N_2_ fixation rates were measured by Shashar et al. (1994b) using the acetylene reduction assay, and following studies suggested that this method is underestimating N_2_ fixation in sponges (Wilkinson 1999). A later study by Mohamed et al. (2008) investigated the diversity and expression of N_2_ fixation genes in bacterial symbionts of four different sponge species from Key Largo, Florida, and suggested that provision of fixed N via the symbionts benefits host sponges in nutrient-limited reef environments. N_2_ fixation by sponge symbionts could therefore be a potentially important source of new N to the reef environment: an assumption that requires further investigation. The correlation between *δ*^15^N signatures of different sponges and the composition of the associated microbial communities (Weisz et al. 2007), together with the evidence of stability of the sponge microbiota over large seasonal shifts (Erwin et al. 2012), strengthen the hypothesis of stable and host-specific associations between bacteria and reef sponges.

Besides sponges, diazotrophs are also found associated with corals (Rohwer et al. 2001, 2002; Frias-Lopez et al. 2002; Lema et al. 2012, 2014) and N_2_ fixation activity has been measured in live hard coral tissues (Williams et al. 1987; Shashar et al. 1994a,b). N_2_ fixation rates detected in corals are comparable to those measured in reef carbonate sediments (Fig. 2). This suggests that their contribution to the input of new N in reef ecosystems may also prove very important when extrapolated to the entire reef area covered by hard substrates.

Endolithic cyanobacteria are common organisms inhabiting the skeleton of scleractinian corals, where they often occur as discrete bands at various depths in the skeletal matrix below the living coral tissue (Le Campion-Alsumard et al. 1995; Fine et al. 2005; Ralph et al. 2007) and can be important in providing nutrients to the coral (Ferrer and Szmant 1988). Recently, evidence of endosymbiosis with N_2_-fixing cyanobacteria in corals was found in the colonial stony coral *Montastraea cavernosa* (Lesser et al. 2004, 2007). Similar symbionts have also been observed in Acroporid corals from the Great Barrier Reef (Kvennefors and Roff 2009), thereby suggesting that this association may be widespread. However, recent studies on different coral species using molecular approaches targeting the *nifH* gene have revealed that diverse diazotrophic assemblages occur associated with coral tissues (Olson et al. 2009; Lema et al. 2012, 2014) and that *nifH*-containing cyanobacteria often represent only a minor fraction of these communities (Lema et al. 2012, 2014). Diazotrophic assemblages in the coral tissue were species specific, with the dominant phylotypes closely related to the bacterial group Rhizobia. Rhizobia species are common soil bacterial symbionts, residing in root nodules of legumes, and function as N_2_ fixers for their host plants. This group was consistently dominant in *Acropora millepora* at different locations throughout the year, suggesting a key functional role also in the coral (Lema et al. 2014).

**Figure 3:**
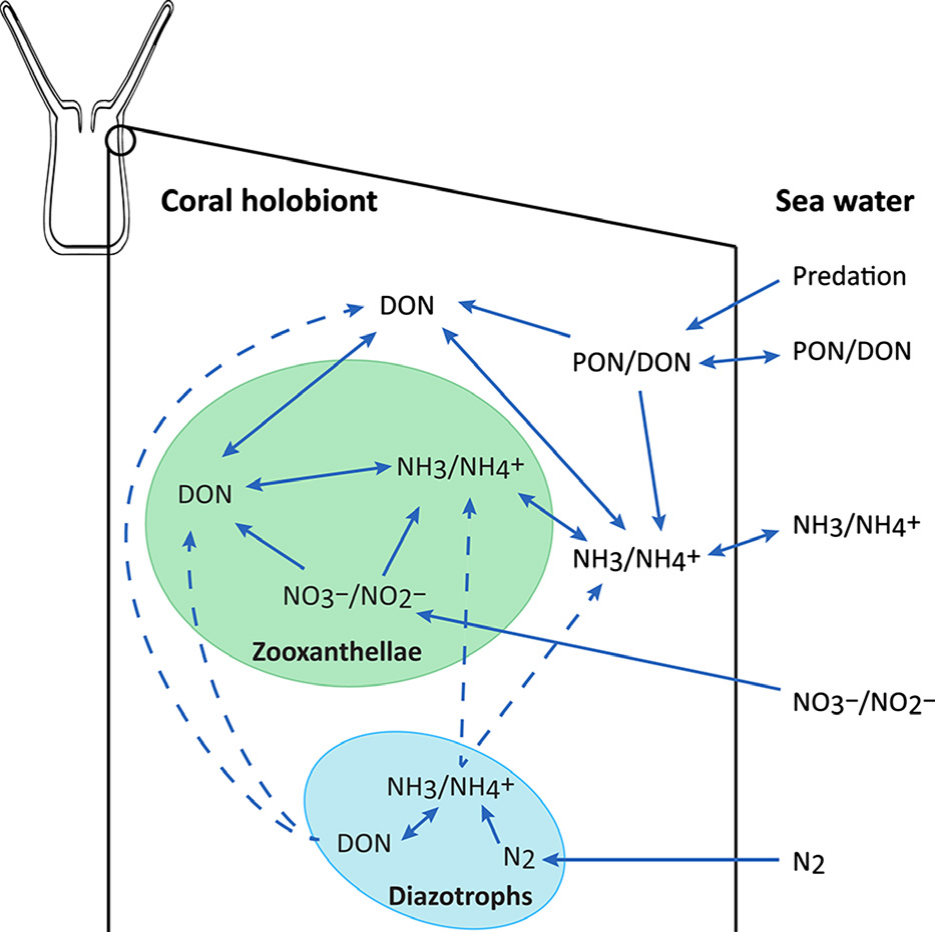
Schematic illustration of the N cycle in the coral holobiont. Solid lines represent nutrient transfer and pathways that have been shown to occur, while dashed lines represent hypothetical fluxes.

Symbiotic corals have evolved a complex internal N cycle which allows them to thrive in N-limited environments (Fig. 3). Both the coral host and its symbiotic dinoflagellate partners (zooxanthellae) possess enzymes enabling rapid ammonium (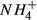) assimilation from the surrounding seawater (Grover et al. 2002; Yellowlees et al. 2008; Stambler 2011; Pernice et al. 2012; Kopp et al. 2013). Moreover, the zooxanthellae are also capable of utilizing nitrate (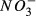) as a nitrogen source (Grover et al. 2003; Kopp et al. 2013). Both the animal tissue and the alga assimilate dissolved organic nitrogen (DON) from the surrounding seawater, with preference to urea and dissolved free amino acids (Grover et al. 2006, 2008; Kopp et al. 2013). Finally, coral polyps are also active particle and zooplankton feeders (Ferrier-Pagès et al. 2003; Mills et al. 2004a).

Nanoscale secondary ion mass spectrometry (NanoSIMS) studies have recently shown that assimilation of both organic and inorganic N sources resulted in rapid incorporation of nitrogen into uric acid crystals, forming temporary N storage sites within the dinoflagellate endosymbionts (Kopp et al. 2013). Another study using a similar methodology showed that coral larvae acquire additional nitrogen (in the form of 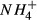) that has been previously taken up from the environment by bacterial partners (Ceh et al. 2013). These results, taken together, draw a picture of corals as opportunistic organisms, which rapidly assimilate and store N from the environment as soon as a source is available.

**Figure 4:**
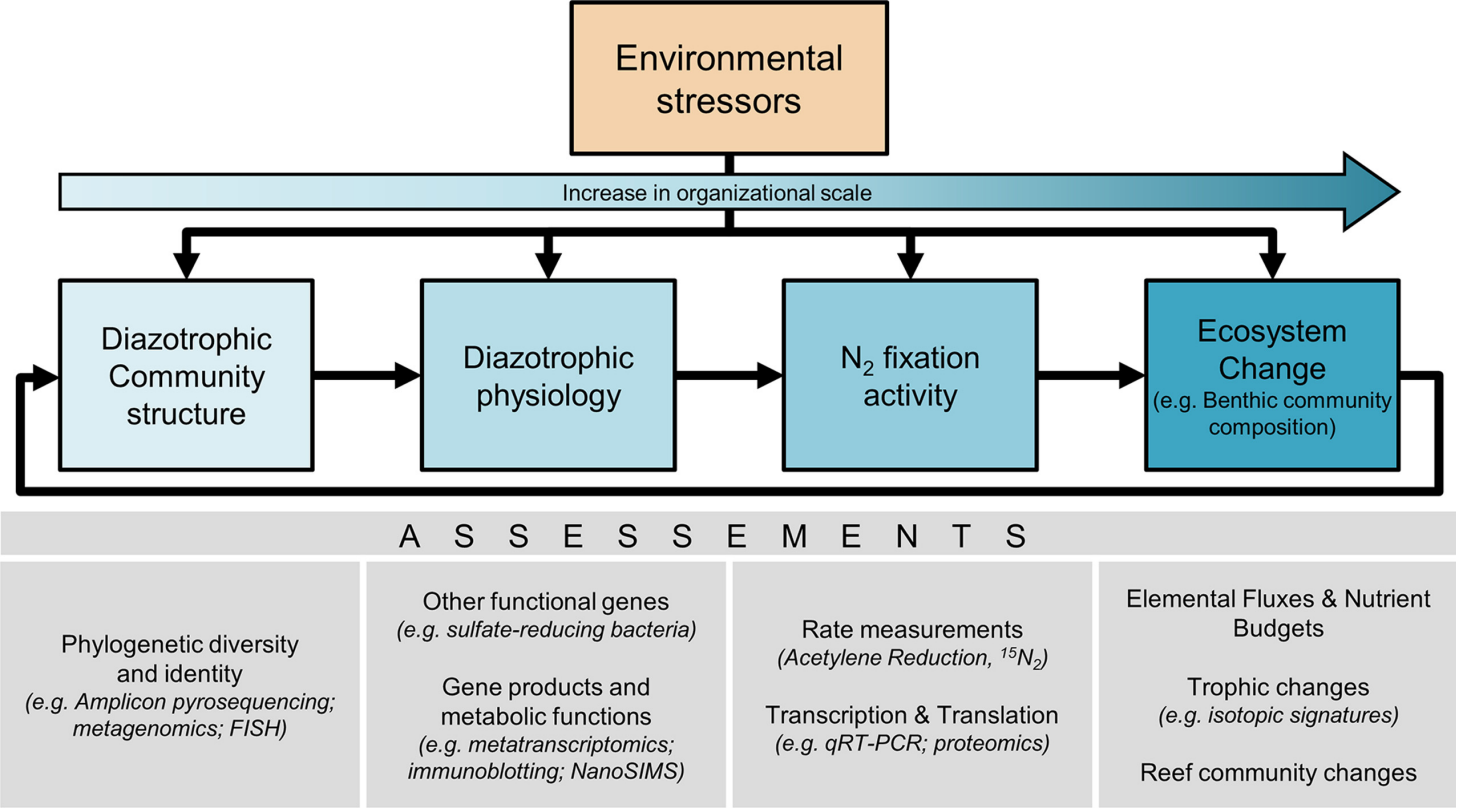
Conceptual diagram showing the structural and functional characterization techniques and approaches useful for assessing environmentally induced changes of the diazotrophic community along various organizational scales, ranging from genome to reef scale.

Other experiments suggest that in coral reef habitats, the growth and abundance of zooxanthellae within the coral host is limited by the availability of dissolved inorganic N (Falkowski et al. 1993). On the other hand, the presence of N_2_ fixers within the host is correlated with higher cell division rate and population size of the endosymbiotic zooxanthellae (Lesser et al. 2007; Olson et al. 2009). Therefore, in the highly N-depleted waters that characterize most coral reefs, the presence of diazotrophs (Fig. 3), thriving in symbiotic association with the corals and their unicellular algae, suggests that N_2_ fixation may be an important additional source of N within the host and may enhance primary productivity. In this multipartner symbiotic system (holobiont) (Knowlton and Rohwer 2003; Krediet et al. 2013), the animal host and the zooxanthellae possibly both benefit from the N fixed by the diazotrophs (Fig. 3), while both the coral and the diazotrophic bacteria receive the photosyntates (i.e., any product of photosynthesis) produced by the dinoflagellate algae. These speculations, together with the approaches and methodologies which have only recently become available (Fig. 4), open an attractive and feasible area of study to identify metabolic interactions among the partners in cnidarian–dinoflagellate–diazotroph symbioses.

As symbioses are widespread in coral reef environments and may be found in a variety of benthic reef organisms other than corals, such as in sponges, mollusks, and foraminifera (Weisz et al. 2010), there is a good chance that benthic N_2_-fixing symbionts are widespread as well. However, further research is needed to examine the likely mutual relationship between C and N_2_ fixers associated with benthic reef organisms that enable reef communities to overcome N limitation. The quantitative importance of N_2_-fixing symbiotic (internal and/or external) associations in coral reef ecosystems is not debated, and we have only begun to investigate the distribution and diversity of diazotrophic populations associated with benthic reef organisms and substrates. Furthermore, the role of diazotrophs in contributing to primary production and growth and their potential susceptibility to climate change still needs to be resolved. As mutualisms (and nutritional mutualisms in particular) bind different species to a common fate, their breakdown as a result of climate change may enhance biodiversity loss and ecosystem disruption (Kiers et al. 2010). It is therefore of the highest priority to investigate the role of N_2_-fixing symbioses in coral reef environments.

## Human-Induced Environmental Changes and their Potential Effects on N_2_ Fixation

### Global warming

There is now a strong body of evidence documenting that earth's climate is changing and that these changes are largely ascribable to human activities (IPCC 2007). In the context of climate change, one of the major consequences affecting the oceans is global warming, which is particularly exacerbated by increasing concentrations of greenhouse gases (mainly carbon dioxide, CO_2_) produced by burning of fossil fuels and deforestation (IPCC 2007). Recent research stresses once more that rigorous and rapid policy decisions are needed in order to save most tropical coral reefs (Frieler et al. 2013) because of their high susceptibility to ocean warming, the latter often resulting in coral bleaching (i.e., the loss of their photosynthetic dinoflagellate endosymbionts) and subsequent mass mortality (Hoegh-Guldberg 1999).

The process of N_2_ fixation is not intrinsically limited by temperature, and active diazotrophs have been found operating at near freezing temperatures (Bordeleau and Prévost 1994) and at hydrothermal vent fluids (92°C) (Mehta and Baross 2006). However, heterocystous cyanobacteria (i.e., cyanobacteria with specialized cells – heterocysts – protecting nitrogenase from O_2_ inhibition) are rare in warm tropical oceans. It has been suggested that the reduced gas solubility and increased respiration rates in warmer waters make the possession of heterocysts under such conditions disadvantageous (Stal 2009), favoring nonheterocystous forms. Moreover, culture studies with the free-living nonheterocystous cyanobacterium *Trichodesmium* spp. showed enhancement of N_2_ fixation and growth under warmer temperatures (Hutchins et al. 2007; Levitan et al. 2010a). Similar diazotrophs (e.g., other *Oscillatoria*) are widespread in benthic environments, so that increases in N_2_ fixation are likely to appear here as well. Nevertheless, heterocystous cyanobacteria form mats in warm coral reef environments (e.g., *Anabaena*) (Charpy et al. 2012a). Therefore, although high temperatures may represent a physiological constrain to the geographic distribution of certain heterocystous species, others may thrive in the range of temperatures expected for future oceans. Thus, much research is needed on the effects of increased temperatures on benthic diazotrophs and their physiology and activity (Fig. 4) if we want to understand the consequences of global warming on the reef N cycle.

Indeed, rising average sea surface temperatures (SST) resulting from global climate change have the potential to increase the amount of N fixed globally (Karl et al. 2002; Rijkenberg et al. 2011), and particularly in coral reef environments (Paul et al. 2005), due to both increased physiological rates of N_2_ fixation as well as increasing N_2_-fixing cyanobacterial populations (Paerl and Huisman 2008). Global warming may in fact further exacerbate phase shifts from corals to algae in reefs subjected to coral bleaching and related coral mortality providing more space for turf, macroalgae, and filamentous cyanobacteria and less space for coral recruitment (Kuffner and Paul 2004; Kuffner et al. 2006; Hughes et al. 2007). As a result, this may increase suitable substrate for diazotrophy (Davey et al. 2008), particularly because the frequency and extent of mass bleaching events are predicted to increase. Indeed, skeletons of thermally bleached corals showed rates up to 30 times greater than those measured on live corals (Davey et al. 2008), highlighting the potential for a substantial change in N inputs in reef ecosystems which suffered large-scale coral death.

Global warming could also lead to an escalation of harmful cyanobacterial blooms (Paerl and Huisman 2008) of species such as the toxin producing genus *Lyngbya* (Albert et al. 2005; Paul et al. 2005), as well as species that have been linked with black band disease (BBD) in corals, including *Phormidium* and others (Rosenberg and Loya 2004), some of which are capable of fixing N_2_. In the tropics, periods with high SST have been increasing in both frequency and extent worldwide in the past 20 years, consequently increasing the necessity for research aiming to understand the effects of such events on the benthic diazotrophic communities inhabiting coral reefs (Fig. 4), particularly after algal blooms or bleaching events which may provide substrate and conditions for diazotrophy.

Finally, the process of N_2_ fixation by symbiotic diazotrophs associated to reef primary producers and its contribution to C fixation will likely be affected, as the balance between symbiont and host interaction is very sensitive to environmental conditions (Knowlton 2001; Krediet et al. 2013). As internal or external symbionts of specific coral reef organisms, N_2_ fixers may be particularly important for providing nutrients to the host during stressful conditions, such as temperature-induced coral bleaching events, when other symbionts (e.g., zooxanthellae) are lost (Fine and Loya 2002; Vega Thurber et al. 2012). Future research looking at the metabolic interactions in the coral holobiont might therefore incorporate measurements of nitrogenase activity (and gene expression) under different temperatures and bleaching conditions. The latter results could help by revealing whether diazotrophs play a role in the metabolism of the coral holobiont, giving unprecedented insights into their functions in a changing ocean.

### Ocean acidification

Uptake of CO_2_ by the ocean directly alters the seawater carbonate chemistry and results in a reduction in *pH* and carbonate saturation and an increase in dissolved inorganic carbon availability (Caldeira and Wickett 2005). These modifications, collectively referred to as ocean acidification (OA), are predicted to cause multifarious impacts on coral reefs at all levels from the organism to the ecosystem.

Coral reef ecosystems are highly dynamic costal systems naturally subject to a high degree of climatological, physical, and biogeochemical variability resulting in diel and seasonal fluctuations in CO_2_ partial pressure (*p*CO_2_) and seawater *pH* (Hofmann et al. 2011; Massaro et al. 2012). However, the steady increase in atmospheric CO_2_ is already shifting the baseline of seawater *pH* in coral reef habitats toward values at which decreases in calcification can cause entire reef systems to fall below the balance between calcification and erosion (Hoegh-Guldberg 2011). To some degree, reef ecosystems are predicted to react and adapt to these changes in seawater carbonate chemistry, and recent evidence suggests that these adaptations may partially offset the expected changes in seawater *pH* (Andersson et al. 2014). However, it is paramount to understand the effects of OA on reef organisms and ecosystems before losing their biodiversity and functioning, especially because “pristine” reefs are essentially already gone (Knowlton and Jackson 2008).

A wide range of reef organisms have been studied under the *pH*/*p*CO_2_ conditions expected to occur at the end of the century, and the responses are variable. OA effects vary from species to species (Fabricius et al. 2011), but are collectively anticipated to be negative on coral reef ecosystem engineers (Wild et al. 2011), with CO_2_ concentrations above 1000 *ppmv* (parts per million by volume) resulting in bleaching and productivity loss (Anthony et al. 2008). On the other hand, fleshy noncalcifying algae and sea grasses flourish under OA conditions (Fabricius et al. 2011; Porzio et al. 2011) as the additional CO_2_ acts as substrate for photosynthesis. Moreover, the decline in grazers and in calcifying epiphytes increases algae development (Hall-Spencer et al. 2008). These results suggest that coral reef benthic community composition may adapt and change in response to the increase in acidity toward communities dominated by primary producers other than corals. This would determine drastic changes in ecosystem functioning with strong feedback on all reef biogeochemical cycles and specifically the N cycle and N_2_ fixation (Fig. 4). In this context, future OA research should focus on the effects of changes in benthic community structure on the associated diazotrophs, their activity, and overall contribution to new N on the reef. Studies addressing this issue may exploit natural pH gradients such as the one in Papua New Guinea (Fabricius et al. 2011), which can be used as a natural model to study ecosystem-level effects of OA on N_2_ fixation in future coral reef habitats.

However, OA may also impact the microbial community and their physiology directly. Microorganisms comprise the largest diversity and biomass of all marine biota, yet how they may be affected by ocean acidification (OA) remains uncertain (Joint et al. 2011). Recent findings emphasize their high sensitivity to expected near-future *pH* changes and highlight the importance of assessing implications of microbial shifts for host health and coral reef processes (Webster et al. 2012). First results looking at the coral-associated microbes also suggest a drastic change in microbial composition in the coral mucus, tissue, and skeleton under OA conditions (Meron et al. 2011). The physiological effects of increasing *p*CO_2_ on N_2_ fixation have very recently been realized, but research up to now only focused on planktonic diazotrophs and cultured isolates. N_2_ fixation by the filamentous nonheterocystous cyanobacterium *Trichodesmium* spp. responded positively to increased *p*CO_2_ (Barcelos et al. 2007). High *p*CO_2_ levels strongly enhance *Trichodesmium* N_2_ and C fixation rates, along with its filament length and biomass (Hutchins et al. 2007; Levitan et al. 2007; Kranz et al. 2009; Lomas et al. 2012). However, recent findings stressed the role of light intensity in modulating the effects of *p*CO_2_ on the process of N_2_ fixation in *Trichodesmium* (Kranz et al. 2010; Levitan et al. 2010b), with high irradiances reducing the stimulatory effect of elevated *p*CO_2_ on gross N_2_ fixation (Garcia et al. 2011). This suggests a potentially limited effect of OA on N_2_ fixation by similar benthic diazotrophs in light-saturated coral reef habitats. In heterocystous species such as *Nodularia spumigena,* rising *p*CO_2_ had an overall stimulating effect on C and N_2_ fixation, as well as on cell growth (Wannicke et al. 2012), whereas ocean acidification had no effects on N_2_ fixation rates in a natural community of unicellular cyanobacteria (Law et al. 2012). However, in culture, the unicellular cyanobacteria *Crocosphaera watsonii* responded to both light and *p*CO_2_ with a significant negative effect on gross:net N_2_ fixation rates (Garcia et al. 2013), implying enhanced cellular retention of fixed N. Low dissolved iron concentrations may also limit the response to higher *p*CO_2_, as the availability of iron influences N_2_ fixation by affecting the synthesis of the Fe-rich proteins of nitrogenase enzyme complex (Kustka et al. 2002; Fu et al. 2008; Shi et al. 2012). As the bioavailability of dissolved Fe is expected to decline because of ocean acidification (Shi et al. 2010), the increase in global oceanic N_2_ and C fixation due to anthropogenic CO_2_ enrichment may be tempered (Fu et al. 2008).

However, it seems clear that OA also has the potential to substantially alter benthic N_2_ fixation, indicated by the finding that *Trichodesmium* N_2_ and C fixation response to elevated *p*CO_2_ was the most pronounced physiological response yet reported for marine microbes (Hutchins et al. 2009; Liu et al. 2010). Similar diazotrophs (i.e., filamentous types) to those found in the open ocean also occur in the benthic environment, particularly in coral reefs, where they may be fundamental in sustaining the high gross primary productivity. Moreover, cyanobacteria are also responsible for deposition of carbonate structures in tropical environments (Steppe et al. 2001), and their study might give precious insight into the effects of OA on benthic diazotrophy. For example, perturbation experiments may be performed looking at the effect of increased *p*CO_2_ on C and N_2_ fixation in these microbial mats. Such studies should also be expanded to other relevant benthic diazotrophs commonly inhabiting coral reefs. These investigations would help in understanding the consequences of increasing anthropogenic CO_2_ on the process of N_2_ fixation in coral reef ecosystems (Fig. 4).

### Ocean eutrophication

During the past century, humans have significantly altered the balance between new N inputs and N losses in the marine environment through the extensive use of synthetic N fertilizers in agriculture, fossil fuel combustion, and coastal urbanization (Codispoti et al. 2001; Schlesinger 2009). Over this time frame, terrigenous discharge and atmospheric N emissions have increased 10-fold and continue to grow as human development expands in coastal watersheds (Howarth et al. 1996). This in turn has the potential to affect N_2_ fixation activity and community composition of diazotrophs in the marine environment.

The high energetic costs associated with N_2_ fixation have resulted in the idea that this process will be suppressed as soon as N compounds are sufficiently available in the surrounding water. However, experimental work demonstrated that N_2_ fixation still occurs at high concentrations of ambient nitrate (Mulholland et al. 2001; Voss et al. 2004; Holl and Montoya 2005; Moisander et al. 2010; Sohm et al. 2011a,b; Großkopf and Laroche 2012), and recent work detected high N_2_ fixation rates associated with sediments from an eutrophic estuary affected by groundwater discharge (Rao and Charette 2012). This may be attributed to enhanced heterotrophic N_2_ fixation due to organic substrate availability promoting oxygen (O_2_) consumption and protecting the enzyme nitrogenase from inactivation (Rao and Charette 2012).

Several studies revealed that nutrients other than N, such as dissolved inorganic phosphate (DIP) and dissolved organic matter (DOM) as well as trace metals (e.g., Fe, Mo), are mostly limiting N_2_ fixation in the open ocean (Wu et al. 2000; Kustka et al. 2002; Mills et al. 2004b; Arrigo 2005; Moutin et al. 2005) and may therefore stimulate N_2_ fixation in coastal areas when supplied from terrestrial sources and anthropogenic inputs. For example, in the Great Barrier reef lagoon, N_2_ fixation by planktonic cyanobacteria (*Trichodesmium*) significantly increased since the 1920s, most likely due to the increased input of river-borne nutrients (e.g., DIP, Fe, DOM) (Bell et al. 1999). Moreover, net primary production in shallow tropical carbonate systems such as coral reefs is often P limited rather than N limited (Smith 1984), but may become N limited as anthropogenic nutrient enrichment accelerates and rates of sediment P adsorption decrease (Howarth et al. 1995; Howarth and Marino 2006). This change might allow diazotrophic microorganisms to proliferate, rather than suppress N_2_ fixation.

Indeed, one of the major threats associated with marine eutrophication is the proliferation and expansion of cyanobacterial harmful algal blooms (Paerl 1997; Paerl and Huisman 2009). As cyanobacteria have a high flexibility in exploiting various N sources (through N_2_ fixation and uptake of organic or inorganic N compounds), their ability to fix N_2_ is advantageous over non-N_2_-fixing phytoplankton species, especially under N-limiting conditions (O'Neil et al. 2012). Marine cyanobacterial blooms (e.g., *Lyngbya, Trichodesmium,* or *Synechococcus*) can significantly alter the competition within the phytoplankton community, thereby further threatening the stability and functioning of the whole ecosystem (O'Neil et al. 2012). Furthermore, the high algal biomass during blooms can decrease benthic light availability, while its subsequent microbial decomposition significantly reduces oxygen availability near the water–sediment interface. Oxygen depletion in bottom waters affects nutrient cycling within the hypoxic zone as less nutrients are retained by the sediment and high amounts of phosphorus and trace metals (iron and molybdenum) are released to the water column (O'Neil et al. 2012). This can further stimulate the growth of planktonic N_2_-fixing cyanobacteria, thereby enhancing the effects of eutrophication (Conley et al. 2009). At the same time, the role of heterotrophic N_2_-fixing bacteria is not well understood and needs to be elucidated. These bacteria can fix N_2_ in organic-rich, anoxic sediments even in the presence of large amounts of ammonium (Howarth et al. 1988), and in the case of SRBs, their N_2_ fixation rates were positively correlated with plankton bloom activities (Bertics et al. 2013).

In coral reef oligotrophic environments, characterized by high light levels reaching the bottom, symbiotic or epiphytic cyanobacteria associated with benthic organisms (e.g., sea grasses, corals) often fix N_2_ at high rates and may significantly contribute to the N inputs (Carpenter and Capone 2008). Research up to now has largely focused on the effects of elevated nutrient concentrations on N_2_ fixation activity by pelagic cyanobacteria (reviewed in Carpenter and Capone (2008)). Nevertheless, in the ENCORE (The Effect of Nutrient Enrichment on Coral Reefs) program (Koop et al. 2001), addition of inorganic N had negative impacts on N_2_ fixation in reef sediments, while inorganic P addition caused a strong increase in N_2_ fixation. Indeed, Koop et al. (2001) suggested N_2_ fixation as a potential biological indicator of nutrient stress in coral reefs, because of the clear and marked response of this variable to the treatments. P enrichement also stimulated rhizosphere N_2_ fixation in the tropical sea grass *Syringodium filiforme*, along with its growth and biomass (Short et al. 1990). Another study investigated the effects of a mass coral spawning event on the N cycle in carbonate reef sediments and found a rapid increase in benthic production when more N became available, reflecting strong short-term N limitation (Eyre et al. 2008). However, coral reef ecosystems may ultimately be P limited because N can be replenished via N_2_ fixation in the longer term (Eyre et al. 2008). As coral reefs are highly susceptible to nutrient inputs due to their proximity to coastal areas (Pastorok and Bilyard 1985) and the balance of symbiont–host interactions can be very sensitive to changing environmental conditions, changes in N_2_ fixation activity following eutrophication will affect N cycles on both the organism and ecosystem level (Fig. 4). Therefore, these hypotheses deserve to be investigated by future research, which should focus on the effects of anthropogenic nutrient inputs on N_2_ fixation by diazotrophs associated with benthic reef organisms and the coral reef ecosystem. Manipulative experiments may help disentangle the effects of the different forms of inorganic and organic P and N on these microbes, on their physiology and activity and therefore on the potential effects on coral reef nutrient cycles.

### Ocean deoxygenation

A major consequence of global climate change, which has only recently received consideration, is the decrease in the dissolved O_2_ content in the World's oceans (Keeling et al. 2010). This phenomenon, called “deoxygenation,” is produced as a result of both the decrease in solubility of O_2_ and the increased upper ocean stratification due to global warming, the latter reducing in turn the O_2_ supply to the ocean interior (Sarmiento et al. 1998; Keeling and Garcia 2002). Additional O_2_ loss caused by eutrophication-induced stimulation of microbial respiration is likely to worsen the problem (Breitburg et al. 2009; Conley et al. 2009).

Coral reefs thrive in shallow well-mixed oxygenated waters and are dominated by photosynthetic organisms. These ecosystems are therefore generally believed to be exempt from the effects of deoxygenation. However, episodic events have been recorded during which dissolved oxygen (DO) levels dropped to hypoxic conditions as a consequence of coral spawning events (Simpson et al. 1993) or phytoplankton blooms (Guzmán et al. 1990), eventually causing extensive mortality of corals and other reef animals over wide areas. Moreover, coral reef benthos naturally experiences strong shifts in DO concentrations on a diel basis as the community shifts from net photosynthesis during the day to respiration during the night. A study from a coral reef platform on the Great Barrier Reef showed that DO can range from 2.1 mg O_2_/L after midnight to more than 10.8 mg O_2_/L (the limit of the instrument) in the early afternoon (Kinsey and Kinsey 1967). Other studies looked at DO levels in the diffusive boundary layer surrounding stony corals and found extreme diel fluctuations, with concentrations varying from supersaturation during the day to anoxia at night (Shashar et al. 1993; Kühl et al. 1995). A later study demonstrated that the presence of sleep-swimming fishes inside coral heads may indeed be considered a mutualism where the fishes find refuge from predation while mitigating hypoxia in the coral branches (Goldshmid et al. 2004). Finally, hypoxic zones occur at the competing interface between algae and corals (Smith et al. 2006; Barott et al. 2009, 2012; Wangpraseurt et al. 2012). Thus, hypoxia has been suggested to play a significant role in coral tissue mortality during coral–algae interaction processes (Haas et al. 2014). As human impacts cause a global decrease in oxygen availability in the water column, eutrophication increases and algae become more abundant on reefs, temporally and spatially restricted hypoxic conditions in coral reefs may become more common. Therefore, research would benefit from studies looking at the alterations of microbial-driven biogeochemical processes occurring in hypoxic zones of coral reefs, which are likely to further expand in the future. In this context, studying diazotrophs and their physiology (Fig. 4) in coral reef hypoxic microenvironments such as coral–algae interactions or looking at pulse ecosystem-level hypoxic conditions on the input of fixed N on the reef may give insights into the effects of decreased oxygen availability on marine diazotrophy.

Generally, low O_2_ levels favor nitrogenase activity as this is irreversibly inhibited by molecular O_2_ (Berman-Frank et al. 2003). Many diazotrophs are only active under anaerobic conditions; others respire to draw down O_2_ levels, or bind O_2_ with proteins such as leghemoglobin (Wittenberg et al. 1974; Robson and Postgate 1980). Under anoxic conditions (no O_2_ present), the microbial community tends to be dominated by SRBs, particularly when nitrate is exhausted (Keeling et al. 2010). Among the microorganisms in the benthos, SRBs can fix N_2_ in a variety of benthic habitats (Nielsen et al. 2001; Steppe and Paerl 2002; Bertics et al. 2010, 2012) and may facilitate N_2_ fixation in sediments beneath hypoxic waters (Bertics et al. 2013). SRBs are important members of the diazotrophic community in sea grass rhizosphere sediments (Capone 1982; McGlathery et al. 1998) as well as in sediments colonized by macroalgae of the genus *Caulerpa* (Chisholm and Moulin 2003).

In summary, these results imply a significant effect of decreasing O_2_ concentrations on the process of N_2_ fixation. Future research efforts should concentrate on the activity of SRBs in coral reef habitats and on their potential role in hypoxic sediments and coral–algae interactions. As O_2_ supply is decreasing in warmer climates, and coastal hypoxia is increasing in the global coastal zone, where it is recognized as a major threat to biota (Steckbauer et al. 2011), major changes are also likely to occur in diazotrophs associated with coral reefs, particularly because several reefs are subjected to a steady coastal influence.

### Ultraviolet radiation (UVR) stress

Anthropogenic inputs of chlorinated fluorocarbons and the consequent decrease in stratospheric ozone have already led to an increase in the amount of harmful ultraviolet radiation (UVR) reaching the biosphere (Stolarski et al. 1992; Kerr and McElroy 1993; Madronich et al. 1998). Furthermore, increasing CO_2_ atmospheric concentrations and resulting climate change will deeply alter the tropospheric ozone budget and increase the ultraviolet index, which would have further consequences for the health and functioning of marine ecosystems (Hegglin and Shepherd 2009).

UVR can penetrate to significant water depths in marine and aquatic ecosystems and may determine significant biological effects on marine biota (Tedetti and Sempéré 2006; Lesser 2008). Moreover, the low solar zenith angle and the natural thinness of the ozone layer over tropical latitudes together with the high transparency of the water column result in the high UVR irradiances that marine organisms experience in shallow-water tropical coral reef environments (Shick et al. 1996; Banaszak and Lesser 2009).

A large body of evidence is available demonstrating the direct and indirect effects of UVR (e.g., DNA damage, photooxidative stress, bleaching, and detrimental effects on reproduction and on larval development) on corals and other reef-associated biota (reviewed in (Banaszak and Lesser 2009). However, studies looking at the effects of UVR on the process of N_2_ fixation only focused on planktonic cyanobacteria. The consequences of increasing UVR reaching the benthos in coral reefs and their associated N_2_ fixers are still to be investigated. Therefore, future research needs to give more attention to the consequences of UVR on benthic reef diazotrophs, particularly because even small anthropogenic increases in UVB levels will have sublethal physiological manifestations in coral reef macroorganisms (Shick et al. 1996).

Ultraviolet radiation, both UVA (320–400 nm) and UVB (290–320 nm), can alter photosynthesis and growth in cyanobacteria (Vincent and Roy 1993). However, cyanobacteria have developed several defense mechanisms helping them to successfully grow and survive in several habitats receiving high solar UVR (Singh et al. 2010) such as coral reefs. N_2_ fixation is also suppressed either directly or indirectly by UVB radiation (Singh et al. 2010) due to the extreme sensitivity of the nitrogenase enzyme (Tyagi et al. 1992; Kumar et al. 2003; Lesser 2008). A 57% decline in N_2_ fixation occurred in cultures of *Anabaena* sp. exposed to UVR, despite an increase in both the concentration of UV photoprotectants and the activity of antioxidant enzymes (Lesser 2008), while several rice-field cyanobacteria showed complete loss of nitrogenase activity (Kumar et al. 2003). These results show that N_2_-fixing cyanobacteria are particularly affected by UVR (Lesser 2008). UVR effects on this group of prokaryotes or on other N_2_-fixing microorganisms may therefore deeply affect the input of new N and the biogeochemical cycling of this essential macronutrient in the oceans. However, UVR may also determine indirect effects on the process of N_2_ fixation in coral reefs. For example, UVR can cause mass coral bleaching and following coral mortality across wide reef areas (Drollet et al. 1995). These mortality events, in turn, provide free space on dead coral colonies, which is often colonized by turf algae and cyanobacterial mats (Davey et al. 2008). Changes in the benthic community composition of coral reefs will consequently have cascading effects on the benthos-associated microbes and their activity (Fig. 4), therefore potentially altering the inputs of new N at the ecosystem scale. As these ecosystems are already subjected to high UV irradiances, which are expected to further increase in the future, the effects of UVR on benthic reef N_2_ fixation deserve attention.

## Conclusions and Perspectives

The functioning of coral reefs is strongly connected with the maintenance of the oligotrophic conditions in which these ecosystems thrive. Variations in the inputs of nutrients to the reef will perturb the tightly coupled recycling and biogeochemical cycles of reef ecosystems, with consequences that are far from being understood. Recent evidence demonstrated that increases in dissolved inorganic nitrogen concentrations decrease the thermal tolerance of corals and increase their susceptibility to bleaching (Wooldridge 2009; Wiedenmann et al. 2013; Vega Thurber et al. 2014). On the other hand, turf and macroalgae are favored under high nutrient availabilities (Jessen et al. 2013). Therefore, these changes may eventually result in phase shifts from coral to algae-dominated communities. It is therefore of the highest priority to investigate and understand the relevance of N_2_ fixation to the whole reef N cycle, in order to predict the effects of human interference in coral reef ecosystems.

Although associations with N_2_-fixing microbes occur in several benthic reef organisms, we currently lack a full understanding of the benefits and costs in many of these associations. There are no studies investigating the abundance and distribution of mutualistic interactions between C and N_2_ fixers in reef organisms, which may significantly contribute to the overall C and N_2_ fixation within the coral reef. As primary productivity is mostly N-limited, this additional source of N from a symbiotic partner represents a further adaptation of these organisms to flourish in oligotrophic reef waters. Gaps of knowledge are present in how N_2_ fixation by benthic diazotrophic reef associations will respond to global climate change and to the increasing anthropogenic CO_2_ dissolving into the oceans. Interacting and synergistic effects of global stressors with local disturbances, such as industrial pollution, sewage and land runoff, dredging, overfishing, and destructive fishing, have scarcely been studied (Harnik et al. 2012; Ateweberhan et al. 2013) but will be essential to understanding and predicting coral reef biogeochemical cycles under conditions of global change.

This article highlights the paramount role of N_2_ fixation in coral reef ecosystems and the vast scope of global environmental impacts that are predicted to affect diazotrophy in future reef habitats. As the reef paradox still needs to be fully resolved, N_2_ fixation in coral reefs is a topic of the highest priority if we want to understand their functioning before the impact of human alterations will deprive us of a baseline for pristine ecosystems (Knowlton and Jackson 2008). In order to understand potential effects within a reasonable timeline, we suggest several high-priority topics: (1) to identify ecological distribution patterns of important benthic diazotrophs; (2) to expand the focus to internal and external associations of N_2_ fixers with benthic eukaryotic organisms; (3) to investigate the potential effects of global and local environmental stressors on the metabolic activity and stability of these partnerships; and (4) to scale the effects of environmental stressors on the input of fixed N to the entire reef ecosystem. Moreover, careful site selection should be considered, where we can utilize natural habitats which currently undergo similar climate-driven stressor(s) expected for the future, for example, CO_2_ seeps.

Given the difficulty in isolation and cultivation of microorganisms, including symbiotically associated types, several culture-independent approaches (Fig. 4) are useful for investigating diazotrophic community structure and metabolic functions in coral reef habitats. Finally, if we want to scale the consequences of changing microbial communities to the effects on the reef ecosystem level, it is essential that we use a multidisciplinary approach. Bridging the gaps among molecular ecology, biochemistry, physiology, and coral reef ecology will be important, as physiological measurements of N_2_ fixation rates as well as ecological and biogeochemical approaches are necessary for understanding the functioning of such a complex ecosystem.

## Supporting Information

**Table S1.** Values in this Table have been collected from the available literature and used to produce the graph in Fig. 3. Rates of N2 fixation have been extrapolated from the text when possible, and the original conversion factor used by the authors has been reported. If no conversion was available, but only C2H2 reduction rates were reported, the conservative 4:1 conversion ratio has been used. ARA = Acetylene Reduction Assay, 15N2 = labeling incubations with isotope 15N2. Only studies with rates normalized to surface area are shown.
