# Supplementary material for: Benthic N2 fixation in coral reefs and the potential effects of human-induced environmental change
Source: Ecol Evol. 2014 Mar 31;4(9):1706–27. doi: 10.1002/ece3.1050 (PMC4063469; doi:10.1002/ece3.1050)
Supplement: Supplementary file 1 [file ece30004-1706-SD1.pdf]

Benthic N_2 fixation in coral reefs and the potential effects of human-induced environmental change

Ulisse Cardini[1*], Vanessa Bednarz[1], Rachel A. Foster[2,4] and Christian Wild[1,3]

[1]Coral Reef Ecology Group (CORE), Leibniz Center for Tropical Marine Ecology (ZMT), Fahrenheitstr. 6, 28359 Bremen, Germany
[2]Max Planck Institute for Marine Microbiology, Celsiusstr. 1, 28359 Bremen, Germany
[3]University of Bremen, Faculty of Biology and Chemistry (FB 2), Bremen, Germany
[4]current address: Department of Ecology, Environment, and Plant Sciences, Stockholm University, SE-10691 Stockholm, Sweden

*corresponding author:
Ulisse Cardini
Tel: +49(0)421/238 00-138
Fax: +49(0)421/238 00-30
Email: ulisse.cardini@zmt-bremen.de

Supplementary Information

Supplementary Table 1. Values in this Table have been collected from the available literature and used to produce the graph in Fig. 3. Rates of N_2 fixation have been extrapolated from the text when possible, and the original conversion factor used by the authors has been reported. If no conversion was available, but only C_2H_2 reduction rates were reported, the conservative 4:1 conversion ratio has been used. ARA = Acetylene Reduction Assay, $^{15}N_2$ = labeling incubations with isotope $^{15}N_2$. Only studies with rates normalized to surface area are shown.

**Substrate**	**Methods**	**Ratio (mol C_2H_2:1 mol N_2)**	**N_2 fixation rates (mg N m^{-2} day^{-1})**	**Location**	**References**
Scleractinian corals	ARA	4.00	3.32 ± 3.70	Eilat, Israel	Shashar *et al.* (1994b)
	ARA	4.00	0.29 - 1.13	Great Barrier Reef, Australia	Davey *et al.* (2008)
	ARA	3.45	9.75 ± 8.18	Eilat, Israel	Shashar *et al.* (1994a)
	ARA	4.00	2.90 ± 2.19	Eilat, Israel	Shashar *et al.* (1994a)
	ARA	4.00	3.69 ± 1.60	Eilat, Israel	Shashar *et al.* (1994a)
	ARA	4.00	5.24 ± 0.25	Eilat, Israel	Shashar *et al.* (1994a)
	ARA	4.00	3.69 ± 4.96	Eilat, Israel	Shashar *et al.* (1994a)
	ARA	3.45	7.21 ± 2.69	Eilat, Israel	Shashar *et al.* (1994a)
	ARA	3.45	0.68 ± 0.45	Eilat, Israel	Shashar *et al.* (1994a)
	ARA	3.00	0.00 - 8.40	Akumal, Mexico	Lesser *et al.* (2007)

Other cnidarians	ARA	4.00	30.0 ± 11.93	Eilat, Israel	Shashar *et al.* (1994b)
	ARA	4.00	0.08 ± 0.25	Eilat, Israel	Shashar *et al.* (1994b)
Sponges	ARA	4.00	0.19 ± 0.34	Eilat, Israel	Shashar *et al.* (1994b)
Macro/Turf Algae	ARA	3.45	0.44 - 1.07	Great Barrier Reef, Australia	Larkum *et al.* (1988)
	ARA	3.45	8.18 ± 12.05	St. Croix, US Virgin Island	Williams and Carpenter (1997)
	ARA	4.00	22.69 ± 9.24	Kaneohe Bay, Oahu, Hawaii	Williams and Carpenter (1998)
	ARA	3.45	10.36 ± 0.56	Eilat, Israel	Shashar *et al.* (1994b)
Limestone/Dead corals	ARA	4.00	0.00 - 42.16	Great Barrier Reef, Australia	Wilkinson *et al.* (1984)
	ARA	4.00	0.51 ± 0.78	Eilat, Israel	Shashar *et al.* (1994b)
	ARA	3.45	10.72 ± 11.30	Great Barrier Reef, Australia	Larkum *et al.* (1988)
	ARA + $^{15}N_2$	3.30	2.12	Tuamotu Atoll, French Polynesia	Charpy-Roubaud *et al.* (2001)
	ARA	3.45	1.05 - 25.98	Great Barrier Reef, Australia	Davey *et al.* (2008)
	ARA	3.45	3.61 - 15.98	Great Barrier Reef, Australia	Larkum (1988)
	ARA	3.45	62.23 ± 31.94	Eilat, Israel	Shashar *et al.* (1994b)
	ARA	3.45	4.68 - 30.79	Great Barrier Reef, Australia	Larkum (1988)
Carbonate sands	ARA	3.00	0.06 - 0.26	Great Barrier Reef, Australia	Werner *et al.* (2008)
	ARA + $^{15}N_2$	3.00	1.80	Barbados	Patriquin and Knowles (1975)
	ARA	4.00	1.10 - 8.50	Great Barrier Reef, Australia	Wilkinson *et al.* (1984)
	ARA	3.00	0.30 - 2.40	Puerto Rico	Corredor and Capone (1985)
	ARA	4.00	0.13 - 4.08	Bermuda	O'Neil and Capone (1989)
	ARA	4.00	0.71 - 5.17	Puerto Rico	O'Neil and Capone (1989)
	ARA	4.00	0.07 - 0.69	San Salvador, Bahamas	O'Neil and Capone (1989)
	ARA	4.00	0.20 - 1.30	Great Barrier Reef, Australia	O'Neil and Capone (1989)
	ARA + $^{15}N_2$	3.16	2.00 - 10.00	Moreton Bay, Australia	O'Donohue *et al.* (1991)
	ARA	3.00	1.68 - 4.37	Great Barrier Reef, Australia	Capone *et al.* (1992)
	ARA + $^{15}N_2$	1.80 - 3.00	0.40 - 3.90	Tuamotu Atoll, French Polynesia	Charpy-Roubaud *et al.* (2001)
	ARA	3.45	21.87 ± 19.61	Eilat, Israel	Shashar *et al.* (1994b)
	ARA + $^{15}N_2$	3.45	0.34 ± 0.09	Great Barrier Reef, Australia	Larkum *et al.* (1988)

	ARA + $^{15}N_2$	3.45	0.81 ± 0.30	Great Barrier Reef, Australia	Larkum *et al.* (1988)
	ARA	4.00	8.21 ± 2.70	New Caledonia	Charpy *et al.* (2007)
Microbial mats	ARA	4.00	16.40 ± 5.40	New Caledonia	Charpy *et al.* (2007)
	ARA + $^{15}N_2$	1.60	8.00	Tuamotu Archipelago, French Polynesia	Charpy-Roubaud and Larkum (2005)
	ARA	4.00	110.00	Enewetak Atoll, Marshall Islands	Mague and Holm-Hansen (1975)
Seagrasses	ARA + $^{15}N_2$	3.16	19.00 - 40.00	Moreton Bay, Australia	O'Donohue *et al.* (1991)
	ARA + $^{15}N_2$	4.40	145.00	Great Barrier Reef, Australia	Iizumi and Yamamuro (2000)
	ARA	3.00	16.00 - 47.00	Gulf of Carpentaria, Australia	Moriarty and O'Donohue (1993)
	ARA	3.00	13.00 - 19.00	Gulf of Carpentaria, Australia	Moriarty and O'Donohue (1993)
	ARA	3.00	25.00	Gulf of Carpentaria, Australia	Moriarty and O'Donohue (1993)
	ARA	n.a.	28.00	Jamaica	Blackburn *et al.* (1994)
	ARA	n.a.	0.03	Florida, USA	McRoy *et al.* (1973)
	ARA	3.00	5.00 - 24.00	Biscayne Bay, Florida, USA	Capone and Taylor (1980)
	ARA	3.00	5.10 - 9.00	Bimini Harbour, Bahamas	Capone *et al.* (1979)
	ARA	3.00	27.00 - 140.00	Barbados	Patriquin and Knowles (1972)

References

Blackburn T, Nedwell D, Wiebe W (1994) Active mineral cycling in a Jamaican seagrass sediment. *Marine Ecology Progress Series,* **110**, 233-239.

Capone DG, Dunham SE, Horrigan SG, Duguay LE (1992) Microbial nitrogen transformations in unconsolidated coral-reef sediments. *Marine Ecology Progress Series,* **80**, 75-88.

Capone DG, Penhale PA, Oremland RS, Taylor BF (1979) Relationship Between Productivity and N2 (C2H 2) Fixation in a Thalassia testudinum Community. *Limnology and Oceanography,* **24**, 117-125.

Capone DG, Taylor BF (1980) N2 fixation in the rhizosphere of Thalassia testudinum. *Canadian Journal of Microbiology,* **26**, 998-1005.

Charpy-Roubaud C, Charpy L, Larkum AWD (2001) Atmospheric dinitrogen fixation by benthic communities of Tikehau Lagoon (Tuamotu Archipelago, French Polynesia) and its contribution to benthic primary production. *Marine Biology,* **139**, 991-997.

Charpy-Roubaud C, Larkum AWD (2005) Dinitrogen fixation by exposed communities on the rim of Tikehau atoll (Tuamotu Archipelago, French Polynesia). *Coral Reefs,* **24**, 622-628.

Charpy L, Alliod R, Rodier M, Golubic S (2007) Benthic nitrogen fixation in the SW New Caledonia lagoon. *Aquatic Microbial Ecology,* **47**, 73-81.

Corredor JE, Capone DG (1985) Studies on nitrogen diagenesis in coral reef sands. In: *The Fifth International Coral Reef Congress.* (eds Gabrie C, Toffart JL, Salvat B) pp Page, Tahiti, French Polynesia.

Davey M, Holmes G, Johnstone R (2008) High rates of nitrogen fixation (acetylene reduction) on coral skeletons following bleaching mortality. *Coral Reefs,* **27**, 227-236.

Iizumi H, Yamamuro M (2000) Nitrogen fixation activity by periphytic blue-green algae in a seagrass bed on the Great Barrier Reef. *Japan Agricultural Research Quarterly,* **34**, 69-73.

Larkum AWD (1988) High rates of nitrogen fixation on coral skeletons after predation by the crown of thorns starfish Acanthaster planci. *Marine Biology,* **97**, 503-506.

Larkum AWD, Kennedy IR, Muller WJ (1988) Nitrogen fixation on a coral reef. *Marine Biology,* **98**, 143-155.

Lesser MP, Falcon LI, Rodriguez-Roman A, Enriquez S, Hoegh-Guldberg O, Iglesias-Prieto R (2007) Nitrogen fixation by symbiotic cyanobacteria provides a source of nitrogen for the scleractinian coral *Montastraea cavernosa*. *Marine Ecology Progress Series,* **346**, 143-152.

Mague TH, Holm-Hansen O (1975) Nitrogen fixation on a coral reef. *Phycologia,* **14**, 87-92.

Mcroy CP, Goering JJ, Chaney B (1973) Nitrogen fixation associated with seagrasses. *Limnology and Oceanography,* **18**, 998-1002.

Moriarty D, O'donohue M (1993) Nitrogen fixation in seagrass communities during summer in the Gulf of Carpentaria, Australia. *Marine and Freshwater Research,* **44**, 117-127.

O'donohue MJ, Moriarty DJW, Rae ICM (1991) Nitrogen fixation in sediments and the rhizosphere of the seagrassZostera capricorni. *Microbial Ecology,* **22**, 53-64.

O'neil JM, Capone D (1989) Nitrogenase activity in tropical carbonate marine sediments. *Marine Ecology Progress Series,* **56**, 145-156.

Patriquin D, Knowles R (1972) Nitrogen fixation in the rhizosphere of marine angiosperms. *Marine Biology,* **16**, 49-58.

Patriquin DG, Knowles R (1975) Effects of oxygen, mannitol and ammonium concentrations on nitrogenase [C2H2] activity in a marine skeletal carbonate sand. *Marine Biology,* **32**, 49-62.

Shashar N, Cohen Y, Loya Y, Sar N (1994a) Nitrogen-fixation (acetylene reduction) in stony corals - evidence for coral-bacteria interactions. *Marine Ecology Progress Series,* **111**, 259-264.

Shashar N, Feldstein T, Cohen Y, Loya Y (1994b) Nitrogen-fixation (acetylene reduction) on a coral reef. *Coral Reefs,* **13**, 171-174.

Werner U, Blazejak A, Bird P *et al.* (2008) Microbial photosynthesis in coral reef sediments (Heron Reef, Australia). *Estuarine Coastal and Shelf Science,* **76**, 876-888.

Wilkinson CR, Williams DM, Sammarco PW, Hogg RW, Trott LA (1984) Rates of nitrogen-fixation on coral reefs across the continental-shelf of the central Great Barrier Reef. *Marine Biology,* **80**, 255-262.

Williams SL, Carpenter RC (1997) Grazing effects on nitrogen fixation in coral reef algal turfs. *Marine Biology,* **130**, 223-231.

Williams SL, Carpenter RC (1998) Effects of unidirectional and oscillatory water flow on nitrogen fixation (acetylene reduction) in coral reef algal turfs, Kaneohe Bay, Hawaii. *Journal of Experimental Marine Biology and Ecology,* **226**, 293-316.
